# Dihydrosanguinarine enhances tryptophan metabolism and intestinal immune function via AhR pathway activation in broilers

**DOI:** 10.1186/s40104-025-01220-x

**Published:** 2025-07-04

**Authors:** Yue Su, Miaomiao Wang, Zhiyong Wu, Peng Huang, Jianguo Zeng

**Affiliations:** 1 College of Veterinary Medicine, Shanxi Agricultural University, Taigu, 030801 Shanxi China; 2 Traditional Chinese Medicine Breeding Center of Yuelushan Laboratory, Changsha, 410128 Hunan China; 3 College of Veterinary, Hunan Agricultural University, Changsha, 410128 Hunan China

**Keywords:** Broiler, Gut microbiota, Intestinal immunity, Sanguinarine, Tryptophan metabolism

## Abstract

**Background:**

Tryptophan is essential for nutrition, immunity and neural activity, but cannot be synthesized endogenously. Certain natural products influence host health by modulating the gut microbiota to promote the production of tryptophan metabolites. Sanguinarine (SAN) enhances broiler immunity, however, its low bioavailability and underlying mechanisms remain unclear. This study aimed to decode the mechanisms by which sanguinarine enhances intestinal immune function in broilers.

**Methods:**

Liquid chromatography-tandem mass spectrometry (LC-MS/MS) was employed to identify the main metabolites of sanguinarine in the intestine. Subsequently, equal concentrations of sanguinarine and its metabolites were separately added to the diets. The effects of sanguinarine and its metabolites on the intestinal immune function of broiler chickens were evaluated using 16S rRNA gene amplicon sequencing and tryptophan metabolomics approaches.

**Results:**

We determined that dihydrosanguinarine (DHSA) is the main metabolite of sanguinarine in the intestine. Both compounds increased average daily gain and reduced feed efficiency, thereby improving growth performance. They also enhanced ileal villus height and the villus-to-crypt (V/C) ratio while decreasing crypt depth and upregulating the mRNA expression of tight junction proteins *ZO-1*, occludin and claudin-1. Furthermore, both compounds promoted the proliferation of intestinal *Lactobacillus* species, a tryptophan-metabolizing bacterium, stimulated short-chain fatty acid production, and lowered intestinal pH. They regulated tryptophan metabolism by increasing the diversity and content of indole tryptophan metabolites, activating the aryl hydrocarbon receptor (AhR) pathway, and elevating the mRNA levels of *CYP1A1*, *CYP1B1*, *SLC3A1*, *IDO2* and *TPH1*. Inflammatory cytokines *IL-1β* and *IL-6* were inhibited, while anti-inflammatory cytokines *IL-10* and *IL-22*, serum SIgA concentration, and intestinal *MUC2* expression were increased. Notably, DHSA exhibited a more pronounced effect on enhancing immune function compared to SAN.

**Conclusions:**

SAN is converted to DHSA in vivo, which increases its bioavailability. DHSA regulates tryptophan metabolism by activating the AhR pathway and modulating immune-related factors through changes in the gut microbiota. Notably, DHSA significantly increases the abundance of *Lactobacillus*, a key tryptophan-metabolizing bacterium, thereby enhancing intestinal immune function and improving broiler growth performance.

## Introduction

The essential amino acid tryptophan, being incapable of endogenous synthesis, serves as a crucial role in animal physiology by modulating nutritional metabolism, immune homeostasis, and neuroendocrine functions [1]. Emerging evidence indicates that specific phytochemicals can dynamically reshape gut microbial ecosystems, thereby optimizing host nutrient utilization and concurrently stimulating microbial biosynthesis of diverse Trp-derived metabolites with immunomodulatory properties [2, 3]. For instance, the contribution of aucubin to atherosclerosis involves the augmentation of *Lactobacilli* in the gut, stimulation of indole-3-β-acrylic acid production, which activates of aryl hydrocarbon receptors (AhR) and inhibits the TGF-β/Smad signaling pathway, aiding in the alleviation of atherosclerosis [4]. Similarly, the administration of sinomenine can significantly enhance the levels of indole tryptophan metabolites by promoting the relative abundance of *Lactobacillus*, thereby activating AhR. This activation effectively mitigates joint damage and inflammation in rheumatoid arthritis through regulation of the phosphorylation process involving NF-κB and MAPK [5].

Nevertheless, the therapeutic potential of these bioactive compounds is frequently constrained by their limited systemic bioavailability, raising questions regarding their capacity to modulate immune responses primarily through parental molecular forms. Current evidence suggests that many poorly absorbed phytochemicals undergo extensive biotransformation into bioactive metabolites that often exhibit superior immunomodulatory efficacy compared to their precursor compounds. A paradigmatic example is berberine, which is enzymatically reduced to dihydroberberine by microbial reductases; this metabolite demonstrates enhanced intestinal barrier-protective effects and remarkable efficacy in ameliorating dextran sulfate sodium (DSS)-induced colitis [6, 7]. Notably, the chemical structure of berberine bears resemblance to that of sanguinarine (SAN), which is one of the active monomeric constituents found in the extract [8, 9]. Previous research has demonstrated that sanguinarine’s broad pharmacological properties, including antibacteria [10], anti-inflammatory [11], antitumor [12] and immunostimulatory effects [13]. The structural similarity between the nitrogen-containing rings of tryptophan and sanguinarine allows sanguinarine to competitively inhibit tryptophan decarboxylase activity. This competitive inhibition regulates the normal metabolism of tryptophan, particularly the production of downstream metabolites such as serotonin and indole derivatives. By modulating tryptophan metabolism, sanguinarine can stimulates appetite, and thereby promotes animal growth and development [14]. Although sanguinarine possesses high pharmacological activity, its practical application is hindered by poor intestinal absorption and low bioavailability [15]. In contrast, its metabolite dihydrosanguinarine (DHSA) exhibits significantly enhanced absorption and utilization efficiency in vivo [16]. Therefore, we hypothesize that sanguinarine may modulate the body's immune function through its intestinal conversion to dihydrosanguinarine.

This study identify that dihydrosanguinarine is the primary metabolite of sanguinarine in broilers. To investigate the effects of sanguinarine and dihydrosanguinarine on intestinal immune function, we supplemented broiler diets with equal concentrations of each monomer. We evaluated their impact on intestinal microbiota structure, tryptophan metabolism, and the AhR pathway. By exploring how active ingredients with low bioavailability affect intestinal immune function through their metabolites, we aimed to uncover the underlying regulatory mechanisms. Consequently, our study not only revealed these mechanisms governing intestinal immune function but also provided a novel perspective for understanding how such compounds regulate immune function throughout the body.

## Materials and methods

### Identification of sanguinarine and its metabolites

**Fig. 1:**
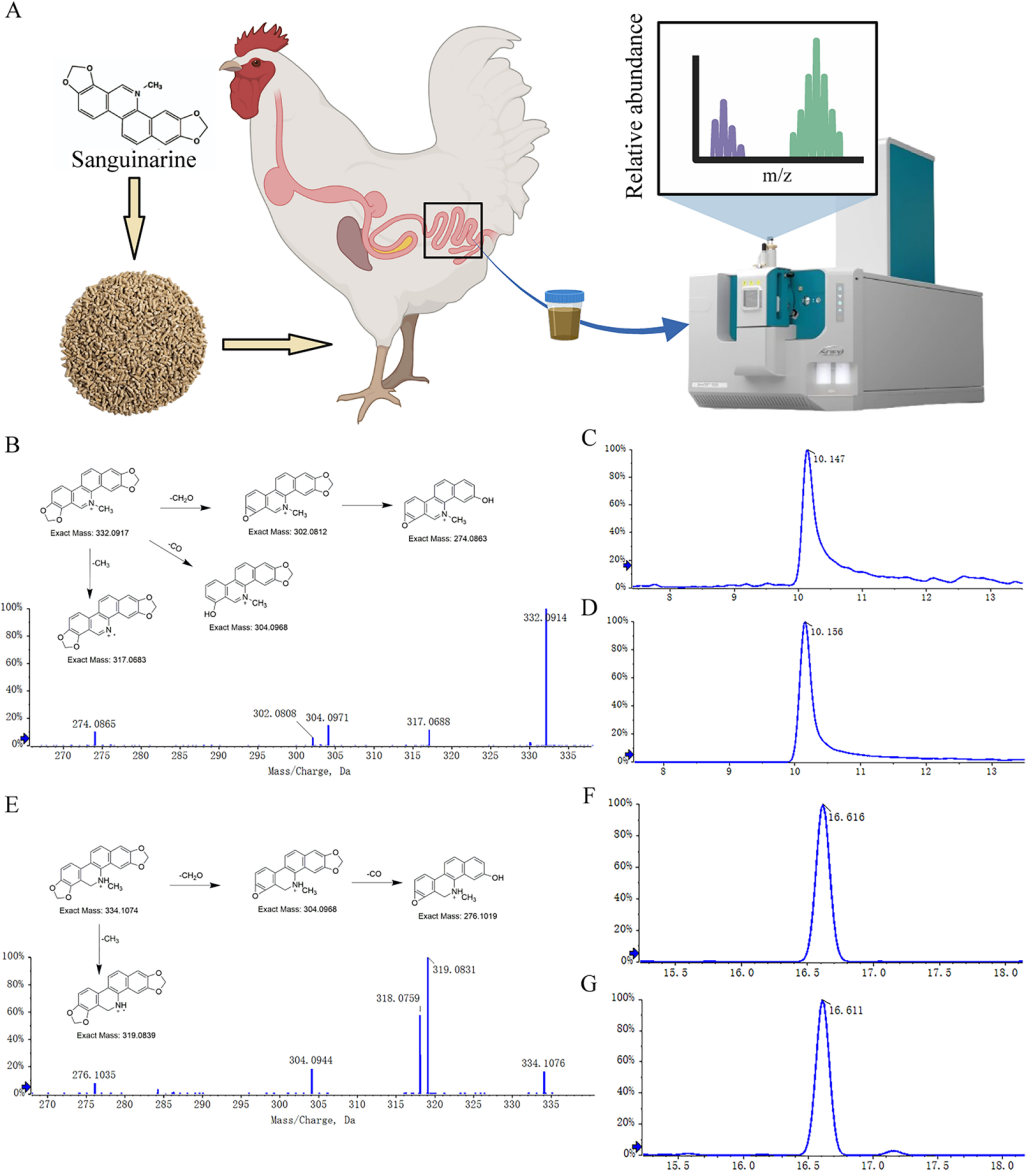
Structural identificati of sanguinarine and its metabolites in the intestine. **A** Schematic diagram of the detection process of sanguinarine metabolites in intestinal contents. **B **MS^2^ chromatogram of M_0_ and fragmentation pathway. **C** Chromatogram of sanguinarine peak emergence time in ileum tissue. **D** Chromatogram of sanguinarine peak emergence time in cecum tissue. **E** MS^2^ chromatogram of M_1_ and fragmentation pathway. **F** Chromatogram of peak emergence time of dihydrosanguinarine in ileum tissue. **G** Chromatogram of peak emergence time of dihydrosanguinarine in cecum tissue

The SAN (purity ≥ 98%, CAS: 140708) used in this experiment was supplied by Hunan Micolta Bioresource Co., Ltd., China, and administered at a dose of 0.225 mg/kg. Samples of ileum and cecum contents (1 g each) were collected from broilers, as depicted in Fig. 1A. Subsequently, 10 mL of acetonitrile was added followed by ultrasonic extraction for 30 min. The resulting mixture was then centrifuged at a speed of 16,000 × *g* for 5 min to obtain the supernatant. Nitrogen drying was carried out at a temperature of 45 °C, and then redissolved by adding 200 μL of methanol and mixing for 2 min. After an additional centrifugation at a speed of 16,000 × *g* for 10 min, the supernatant was collected. Finally, mass spectrometry analysis was performed using the SCIEX Zeno TOF™ 7600 system.

The chromatographic parameters were as follows: SAN and its metabolites were separated using an XAqua column (2.1 mm × 150 mm, 5 μm), which was maintained at a temperature of 35 °C. The mobile phase consisted of 0.1% aqueous solution of formic acid (A) and acetonitrile solution (B). The flow rate was set at 0.3 mL/min with an injection volume of 2 µL. A gradient elution procedure was employed as follows: from 0 to 12 min, the composition changed from 10% to 20% B; from 12 to 15 min, it changed from 25% to 85% B; at 15 to 16 min, it reached 90% B; between 16 to 20 min, it remained constant at 90% B; then decreased gradually from 20 to 21 min to reach 10% B; finally maintaining 90% B until 25 min.

The parameters for the MS and MS^2^ were set as follows: The initial mass spectrometry acquisition were adjusted with nebulizer gas pressure of 50 psi, heating gas pressure of 50 psi, curtain gas pressure of 35 psi, heating gas temperature of 550 °C, collision gas pressure of 8 psi, capillary voltage of 5,500 V, and defragmentation voltage kept constant at 80 V. The mass scanning range (*m/z*) was configured from 100 to 1,000 Da, with the collision energy set to 12 eV and accumulation limited to no longer than 0.15 s. For subsequent mass spectrometry analysis utilizing information-dependent scanning, the collision energy was adjusted to 35 eV, and the accumulation time was reduced to approximately 0.015 s. Meanwhile, the total scanning time was approximately 0.804 s.

### Experimental design

**Fig. 2:**
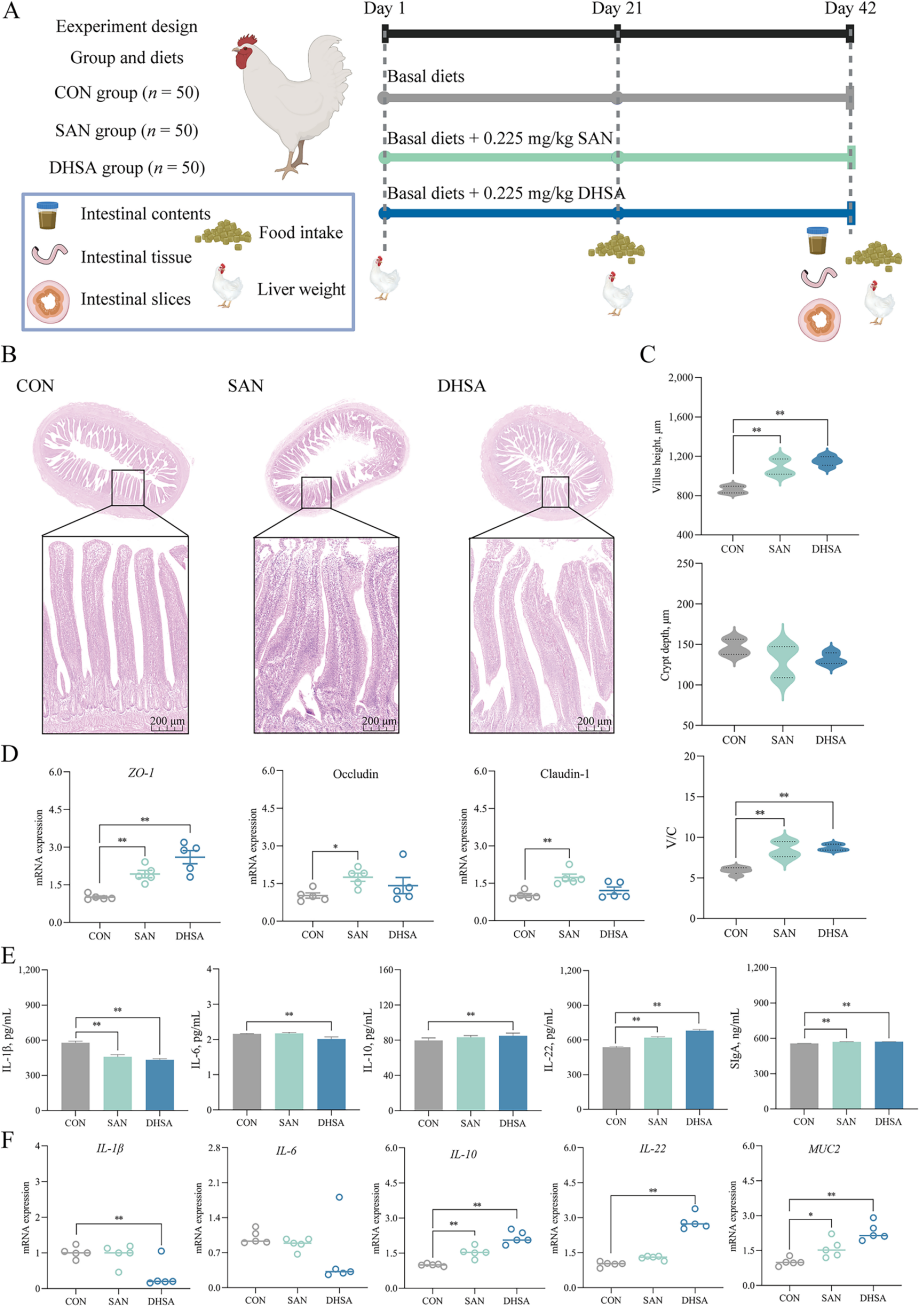
Sanguinarine and dihydrosanguinarine enhanced ileal immune function in broilers. **A** Experimental grouping and treatment. **B** Optical microscopy was used to monitor HE-stained sections. **C** The villus height, crypt depth and villus height / crypt depth (V/C) of the ileum were analyzed using HE staining. **D** The mRNA expression levels of *ZO-1*, occludin and claudin-1 were examined using qRT-PCR. **E** The levels of IL-1β, IL-6, IL-10, IL-22 and SIgA in the ileum were analyzed by ELISA kits. **F** The relative expression levels of *IL-1β*, *IL-6*, *IL-10*, *IL-22* and *MUC2* in the ileum were analyzed by qRT-PCR. CON Control group (basal diet), SAN Sanguinarine group (basal diet containing 0.225 mg/kg sanguinarine), DHSA Dihydrosanguinarine group (basal diet containing 0.225 mg/kg dihydrosanguinarine). Values are expressed as mean ± SEM (*n* = 5). *P < 0.05; **P < 0.01

The experiment utilized a total of 150 broiler chickens with comparable body weight, as illustrated in Fig. 2A. These chickens were sourced from a commercial hatchery (Hunan Shuncheng Industrial Co., Ltd., China), and were equally distributed between males and females. They were randomly assigned to three groups, each consisting of five replicates with 10 chickens per replicate. The groups consisted of the CON group (basal diet), the SAN group (basal diet supplemented with 0.225 mg/kg SAN), and DHSA group (basal diet supplemented with 0.225 mg/kg DHSA). The DHSA (purity ≥ 98%, CAS: 3606-45-9) was supplied by Shanghai Yuanye Bio-Technology Co., Ltd. The experimental period spanned 42 d and was divided into two stages: an early stage (1 to 21 d) and a late stage (22 to 42 d). The formulation of the basal diet followed the guidelines outlined in the China National Feeding Standard for Chicken (NY/T 33-2004), with specific ingredients and nutritional levels provided in Table 1. Following the conclusion of each experimental phase, a 12-h fasting period was observed. Individual body weight and feed intake were meticulously recorded for each group. Utilizing this data, the average daily feed intake (ADFI), average daily gain (ADG), and feed efficiency (FE) were computed for each group.

**Table 1:** Ingredients and nutrient composition of the experimental diets (as-fed basis)

Item	1 to 21 d	22 to 42 d
Ingredients, %		
Corn	55.23	61.00
Soybean meal	36.00	30.00
Soybean oil	4.60	5.20
Limestone	1.20	1.10
DL-Methionine	0.22	0.13
L-Lysine·HCl	0.56	0.30
CaHPO_4_	1.59	1.67
Premix^a^	0.60	0.60
Total	100.00	100.00
Nutrient levels^b^		
Metabolizable energy, kcal/kg	3,023.64	3,116.25
Crude protein, %	19.90	17.78
Crude fiber, %	3.44	3.17
Calcium, %	0.88	0.84
Available phosphorus, %	0.35	0.35
Lysine, %	1.41	1.12
Methionine, %	0.52	0.41
Tryptophan, %	0.25	0.22

^a^Premix provides the following per kg of diet: Cu (as copper sulfate pentahydrate), 4.48 mg; Fe (as ferrous sulfate), 16.58 mg; Zn (as zinc sulfate), 38.13 mg; Mn (as manganese sulfate), 45.11 mg; Se (as sodium selenite), 0.5 mg; I (as potassium iodide), 1.5 mg; multivitamin, 300 mg; choline, 500 mg; antioxidant, 100 mg; zeolite powder, 400 mg; fine bran, 380 mg; phytase, 200 mg; salt, 3,000 mg

^b^Calculated value based on the analysis of experimental diets

### Sample collection

Following the experimental period, five chickens with comparable average body weight were randomly selected from each group. The sections of ileal tissue were excised and preserved in 4% paraformaldehyde solution. Concurrently, the contents of the ileum and cecum were gathered, immediately frozen in liquid nitrogen, and stored at −80 °C for subsequent analyses. The remaining tissues were cleaned, rapidly frozen in liquid nitrogen, and also stored at −80 °C.

### Intestinal morphology

The fixed ileal tissue specimens were dehydrated and embedded in paraffin. Tissue sections (thickness of 5 μm) were stained with hematoxylin and eosin (H&E) and examined using an Olympus BX50 (Tokyo, Japan). Histological evaluations were subsequently performed using an Olympus U-TV0.63XC microscopy (Tokyo, Japan). Statistical analysis was conducted on five randomly selected fields from each section. The CaseViewer software was used to measure the height of each intestinal villus and its corresponding crypt depth and to calculate the ratio of the two values.

### Cytokines and immunoglobulins

Ileum samples (0.1 g) were homogenized in 0.9 mL of phosphate-buffered saline and centrifuged at 3,000 × *g* at 4 °C for 10 min. The resulting supernatants were collected and used to measure the concentrations of interleukin-1β (IL-1β), interleukin-6 (IL-6), interleukin-10 (IL-10), interleukin-22 (IL-22) and secreted immunoglobulin A (SIgA) in broilers were utilized to measure by enzyme-linked immunosorbent assay (ELISA) kits (mlbio, Shanghai, China).

### Tryptophan and its metabolites

The intestinal content samples (*n* = 3) were collected from broilers in each group, and 500 μL of methanol was added. A 20-μL internal standard solution (250 ng/mL) was included for quantification. The extraction solution was vortexed for 3 min and incubated at −20 °C for 30 min. Subsequently, the extraction solution was centrifuged at 4 °C and 16,000 × *g* for 10 min to obtain a supernatant volume of 250 μL. This supernatant underwent an additional centrifugation step under the same conditions for 5 min. Sample extracts were analyzed using an LC-ESI-MS/MS system (UPLC, ExionLC AD; MS, QTRAP^®^6500+ system). A triple quadrupole-linear ion trap mass spectrometer (QTRAP^®^6500+ LC-MS/MS system) equipped with an ESI Turbo ion spray interface was used. The system operated in both positive and negative ion modes and was controlled by Analyst software version 1.6.3. The ESI source parameters were set as follows: ESI^±^ mode; Source temperature set at 550 °C; ion spray voltage set at 5,500 V (positive) and −4,500 V (negative), respectively. The gas pressure was maintained at 35 psi. Tryptophan and its metabolites were analyzed using a multi-reaction monitoring method. The data acquisition was conducted utilizing Analyst software version 1.6.3, while the quantification of all metabolites was performed using Multiquant software version 3.0.3. Principal component analysis was performed in R using the statistical function stats version 3.5.1. Significantly regulated metabolites among groups were determined based on the following criteria: VIP ≤ 1 and Log_2_FC ≥ 1.0 (equivalent to a fold change of ≥ 2). Spearman correlation analysis was used to calculate the correlation between immune-related factors and tryptophan metabolites in intestinal tissues, followed by the construction of a correlation cluster heatmap. All data analyses were conducted on the Metware Cloud online data analysis platform (https://cloud.metware.cn).

### Molecular docking

Previous studies have demonstrated that the expression of intestinal AhR pathway genes in broilers can be regulated by SAN [17]. For specific details, please refer to Table 2. Based on these findings, we selected two genes (*CYP1A2* and *TPH1*) as molecular docking targets and obtained their protein structures from the UniProt database (https://www.uniprot.org/). The 3D structures of small molecule compounds SAN and DHSA were imported in PDF format from PubChem (https://pubchem.ncbi.nlm.nih.gov/). Molecular docking was performed using AutoDock software, and the resulting conformations were analyzed with PyMOL to identify the optimal binding conformation with minimal energy.

**Table 2:** The expression levels of AhR pathway genes in the CON group and the SAN group

Name^a^	CON	SAN	Log_2_FC	*P*-value	Up/Down
*AhR*	30.53	26.69	−0.12	0.472	down
*AhRR*	6.85	7.12	0.15	0.505	up
*HSP90B1*	361.35	390.82	0.10	0.565	up
*ARNT*	63.49	64.89	0.04	0.820	up
*UGT1A1*	269.24	373.31	0.53	0.020	up
*ARNT*	0.95	1.47	0.72	0.139	up
*CYP1A1*	0.61	2.94	2.35	0.012	up
*CYP1A2*	0.43	7.02	3.92	1.000	up
*CYP1B1*	4.19	4.55	0.15	0.669	up
*NQO1*	133.62	162.87	0.16	0.498	up
*TPH1*	2.76	3.18	0.22	0.497	up
*TPH2*	0.06	0.07	0.28	0.880	up
*TDO2*	0.50	0.59	0.29	0.608	up
*IL22*	4.57	5.68	0.25	0.847	up

^a^*AhR* Aryl hydrocarbon receptor, *AhRR* Aryl-hydrocarbon receptor repressor, *HSP90B1* Heat shock protein 90 beta family member 1, *ARNT* Aryl hydrocarbon receptor nuclear translocator, *UGT1A1* UDP glucuronosyltransferase family 1 member A1, *ARNT* Aryl hydrocarbon receptor nuclear translocator, *CYP1A1* Cytochrome P450, family 1, subfamily A, polypeptide 1, *CYP1A2* Cytochrome P450, family 1, subfamily A, polypeptide 2, *CYP1B1* Cytochrome P450, family 1, subfamily B, polypeptide 1, *NQO1* NAD(P)H quinone dehydrogenase 1, *TPH1* Tryptophan hydroxylase 1, *TPH2* Tryptophan hydroxylase 2, *IDO2* Indoleamine 2,3-dioxygenase 2, *IL22* Interleukin 22

### qRT-PCR

Total RNA was extracted from the tissues using an RNA isolation kit supplied by Accurate Biotechnology (Hunan) Co., Ltd. Subsequently, cDNA synthesis and quantitative real-time PCR (qRT-PCR) analysis were performed using the Transcriptor First-Strand cDNA Synthesis Kit and SYBR^®^ Green I Master Mix, both also provided by Accurate Biotechnology (Hunan) Co., Ltd. The relative mRNA expression levels of the target genes were determined using the 2^−ΔΔCT^ method, with β-actin serving as the internal control gene. The amplification primers were designed and synthesized by Tsingke Biotech Co., Ltd. (Beijing, China), and their sequences are listed in Table 3.

**Table 3:** Sequences of the primers used for qRT-PCR

Gene^a^	Primers^b ^(5′→3′)	GenBank accession
β-actin	F: GCACCACACTTTCTACAATGAG	NM_205518.2
	R: ACGACCAGAGGCATACAGG	
*ZO-1*	F: GCCAGCCATCATTCTGACTCCAC	XM_046925214.1
	R: GTACTGAAGGAGCAGGAGGAGGAG	
Occludin	F: TACGGCAGCACCTACCTCAA	XM_046904540.1
	R: AGGCAGAGCAGGATGACGAT	
Claudin-1	F: GCCACGTCATGGTATGGCAA	NM_001013611.2
	R: CCAGCCAATGAAGAGGGCTG	
*IL-1β*	F: CACTGGGCATCAAGGGCTACAAG	NM_204524.2
	R: GTCCAGGCGGTAGAAGATGAAGC	
*IL-6*	F: GAAATCCCTCCTCGCCAATCTGAAG	NM_204628.2
	R: GCCCTCACGGTCTTCTCCATAAAC	
*IL-10*	F: CGCTGTCACCGCTTCTTCA	NM_001004414.4
	R: CGTCTCCTTGATCTGCTTGATG	
*IL-22*	F: CTTCTGCTGTTGTTGCTGTTTCCC	NM_001199614.1
	R: GCCAAGGTGTAGGTGCGATTCC	
*MUC2*	F: ATGCGATGTTAACACAGGACTC	XM_040673076.2
	R: GTGGAGCACAGCAGACTTTG	
*CYP1A1*	F: AGGACGGAGGCTGACAAGGTG	NM_205147.2
	R: AGGATGGTGGTGAGGAAGAGGAAG	
*CYP1A2*	F: CGACCACGACGACCAGGAG	NM_205146.3
	R: GGTAGCGGAGCAGAGGGATG	
*CYP1B1*	F: GTATCCAAAAGTGCAGGCTA	XM_015283751.4
	R: GTGACATTCAAGGTAAACGG	
*IDO2*	F: CCATCCTCACCCACGCAGATTTC	XM_040651540
	R: GGGCTCCAGGTTCTCAATTTCCAG	
*TPH1*	F: CTCGGACCCTCTCTACACACCAG	NM_204956.2
	R: TGGACAGCCTCGTCTGATGCC	
*SLC3A1*	F: AGTGAGAAGGGAGGTGTGGAGAAC	XM_004935370.5
	R: CATCTTGGCTGCTGGTGTCAGTATC	

^a^*ZO-1* Zonula occludens-1, *IL-1β* Interleukin 1 beta, *IL-6* Interleukin 6, *IL-10* Interleukin 10, *IL-22* Interleukin 22, *MUC2* Mucin 2, *CYP1A1* Cytochrome P450, family 1, subfamily A, polypeptide 1, *CYP1A2* Cytochrome P450, family 1, subfamily A, polypeptide 2, *CYP1B1* Cytochrome P450, family 1, subfamily B, polypeptide 1, *IDO2* Indoleamine 2,3-dioxygenase 2, *TPH1* Tryptophan hydroxylase 1, *SLC3A1* Solute carrier family 3 member 1

^b^*F* Forward, *R* Reverse

### 16S rRNA sequencing

The gut microbiota of five broilers was analyzed by isolating microbial DNA from their intestinal contents using the OMG-Soil DNA Kit (Omega Bio-Tek, Georgia, USA). The quantity and quality of the isolated DNA were determined using the QuantiFluor™ ST Blue Fluorescence Quantitation System (Promega) and validated through 1% agarose gel electrophoresis. The V3–V4 region of the bacterial 16S rRNA gene was amplified with primer pairs 338F (5′-ACTCCTACGGGAGGCAGCAG-3′) and 806R (5′-GGACTACHVGGGTWTCTAAT-3′) using an ABI GeneAmp^®^ 9700 thermocycler PCR system. Paired-end sequencing libraries (2 × 300 bp) were generated from the purified PCR products and sequenced on the Illumina MiSeq platform. The raw FASTQ files were demultiplexed using a custom Perl script. The demultiplexed reads were then paired, trimmed, and quality-filtered using fastp version 0.19.6 to remove lower coverage reads, Subsequently, assembled into contigs using FLASH version 1.2.7 for downstream analysis. Operational taxonomic units (OTUs) were delineated at a 97% sequence similarity threshold using the Uparse algorithm. Representative sequences from each OTU were identified and classified for further bioinformatics analysis. The alpha diversity was estimated by calculating Chao index and Shannon index and visualized using boxplots. Beta diversity was estimated by calculating unweighted UniFrac and weighted UniFrac distances and visualized using principal coordinate analysis (PCoA). Kruskal‒Wallis rank sum test was used to analyze differences in bacterial abundances among groups. Spearman’s correlation analysis was employed to explore associations between gut microbiota and other variables. All data processing and analysis were conducted on the Majorbio cloud platform (www.majorbio.com).

### The effect of sanguinarine and dihydrosanguinarine on *Lactobacillus reuteri*

The research methods employed by this laboratory were designed based on a previously established standard protocol [18]. In brief, an initial stock of *Lactobacillus reuteri* (CICC 6119) was purchased from the China Center of Industrial Culture Collection. The stock was subcultured twice in MRS broth containing varying concentrations (3 and 24 ng/mL) of sanguinarine and dihydrosanguinarine. Subsequently, the cultures were streaked onto MRS agar plates and incubated at 37 °C for 24 h. Throughout the experiments, both the OD value and pH value were measured. Growth curves depicting the fermentation time and viable counts every 2 h were constructed for the *Lactobacillus reuteri* group.

### Short-chain fatty acid (SCFA)

The short-chain fatty acid content in the bacterial solution was determined using Gas Chromatography [13]. Briefly, *Lactobacillus reuteri* was cultured in vitro with sanguinarine and dihydrosanguinarine at 37 °C for 24 h. Bacterial fermentation broths (0.9 mL) were transferred to a 2-mL tube and combined with 0.1 mL of 25% metaphosphoric acid. After filtration through a 0.45-μm membrane, the sample was prepared for analysis. The specifications of GC column are DB-FFAP (30 m × 0.25 mm × 0.25 μm). An aliquot of 1 μL from the supernatant was analyzed using a gas chromatograph (Shimadzu 2010 plus type gas chromatogram) equipped with a flame ionization detector (FID). A flame ionization detector was used with an injection port temperature of 250 °C, and a detector temperature of 280 °C, and nitrogen gas was used as the carrier gas at a flow rate of 1.8 mL/min.

### Statistical analysis

Data analysis and processing were performed using SCIEX OS Explorer. Statistical analysis for each group was conducted using SPSS 27.0 software. Variations among groups were assessed using one-way ANOVA, followed by multiple comparisons with Tukey-Kramer test. GraphPad Prism 8.0 was used to generate corresponding plots. Spearman's rank correlation analysis was employed to evaluate the correlations between tryptophan and its metabolites, intestinal immune-related factors, as well as microbial structure and abundance. The results are presented as mean ± standard error of the mean (SEM). A significance level of *P* < 0.05 indicates statistical significance, while *P* < 0.01 indicates a highly significant difference.

## Results

### Structural identification of sanguinarine and its metabolites in the intestine

The mass spectrometry analysis depicted in Fig. 1B–D reveals that M_0_ yields a molecular ion, denoted as [M]^+^, at *m/z* 332.0910. Notably, the peak retention time of M_0_ in both the ileum and cecum of broilers is precisely 10.1 min, consistent with the expected molecular formula C_20_H_15_NO_4_ (Calculated: 332.0917). The measured molecular ion peak exhibits an error range within 5 ppm compared to its theoretical value. Additionally, the MS/MS spectrum demonstrates that the precursor ion at *m/z* 332.0910 undergoes fragmentation by losing CH_3_, CO, CH_2_O and C_2_H_2_O_2_ groups, resulting in product ions with *m/z* values of 317.0668, 304.0954, 302.0802, and 274.0869, respectively. The observed molecular weights and cleavage pattern of these product ions are consistent with those of sanguinarine, thus confirming M_0_’s identification as sanguinarine. As depicted in Fig. 1E–G, metabolite M_1_ generates excimer ions [M + H]^+^ at *m/z* 334.1068. The peak retention time of both ileum and cecum is 16.6 min, aligning with the molecular formula C_20_H_15_NO_4_^+^ (Calculated C_20_H_15_NO_4_^+^: 334.1074). The discrepancy between the measured molecular ion peak and the theoretical value is less than 5 ppm. Fragment ions at *m/z* 334.1068 yield fragments with *m/z* values of 319.0834, 304.0957, and 276.1014 through the loss of CH_3_, CH_2_O, and C_2_H_2_O_2_ groups, respectively. The molecular weights of these product ions are approximately 2 Da higher than those of M_0_, while their fragmentation pathways remain consistent with those observed for M_0_. Consequently, we hypothesize that M_1_ serves as a metabolite derived from M_0_ and identified it as dihydrosanguinarine by comparing its peak retention time and secondary fragment ions with those of the dihydrosanguinarine standard (Fig. S1).

### Sanguinarine and dihydrosanguinarine enhanced ileal immune function in broilers

As demonstrated in Fig. 2B and C, compared with the CON group, dietary supplementation with SAN and DHSA significantly increased villus height in the ileum of broilers (*P* < 0.001). As shown in Fig. 2D, the SAN group exhibited a significant increase in the mRNA levels of *ZO-1* (*P* = 0.003) and claudin-1 (*P* = 0.001), while the mRNA level of occludin was also increased (*P* = 0.035). Moreover, the mRNA expression of *ZO-1* in the ileum of broilers in the DHSA group was extremely significantly increased (*P* < 0.001). As shown in Fig. 2E, all experimental groups of broilers exhibited a significant decrease in the content of IL-1β in the ileal tissue compared to the CON group (*P* < 0.001). Meanwhile, the levels of IL-22 (*P* < 0.001) and SIgA (*P* < 0.001) were significantly increased. Additionally, dietary supplementation with DHSA significantly decreased the concentration of IL-6 in the ileum of broilers (*P* < 0.001), while the content of IL-10 (*P* = 0.008) showed a significant increase. The mRNA expression levels of immune-related factors in the ileal tissues of broilers were determined by qRT-PCR, and the results were shown in Fig. 2F. Compared with the CON group, the mRNA expression levels of *IL-10* in the ileum of broilers in both the SAN and DHSA groups were significantly increased (*P* = 0.002 and *P* < 0.001, respectively). Additionally, dietary supplementation with SAN significantly increased the expression of *MUC2* (*P* = 0.0018) in the ileum of broilers. The DHSA group exhibited a significant increase in the contents of *IL-22* (*P* < 0.001) and *MUC2* (*P* < 0.001), as well as a significant decrease in the expression of *IL-1β* (*P* = 0.004). The intestinal immune status is closely associated with nutrient absorption, metabolic efficiency, and overall health in broilers. Therefore, enhancing intestinal immune function can facilitate the optimal growth and development, as demonstrated in Table 4. During the initial phase of the experiment (1–21 d), compared to the CON group, broilers in the DHSA group exhibited a significant increase in ADG (*P* = 0.049), while broilers in both SAN and DHSA groups showed a significant decrease in FE (*P* = 0.005). During 22–42 d and 1–42 d, supplementation with DHSA significantly increased ADG (*P* = 0.021) and significantly reduced FE values (*P* = 0.008 and *P* = 0.002).

**Table 4:** Effects of dietary sanguinarine and dihydrosanguinarine supplementation on growth performance of broilers

Items	Groups			*P*-value
	CON	SAN	DHSA	
BW, g				
1 d	44.60 ± 0.02	44.60 ± 0.02	44.60 ± 0.02	0.889
21 d	623.80 ± 2.63	635.48 ± 7.53	638.20 ± 2.13	0.054
42 d	2,292.00 ± 14.54	2,329.00 ± 26.71	2,371.20 ± 16.98^*^	0.017
1–21 d				
ADFI, g	38.11 ± 0.27	37.42 ± 0.32	37.88 ± 0.26	0.255
ADG, g	27.58 ± 0.13	28.14 ± 0.36	28.27 ± 0.10^*^	0.049
FE	1.38 ± 0.01	1.33 ± 0.01^**^	1.34 ± 0.01^**^	0.005
22–42 d				
ADFI, g	117.05 ± 1.26	117.17 ± 1.37	114.45 ± 1.87	0.241
ADG, g	79.44 ± 0.74	80.64 ± 1.04	82.52 ± 0.75^*^	0.021
FE	1.48 ± 0.03	1.45 ± 0.01	1.39 ± 0.02^**^	0.008
1–42 d				
ADFI, g	77.58 ± 0.67	77.29 ± 0.80	76.16 ± 0.92	0.220
ADG, g	53.51 ± 0.35	54.39 ± 0.64	55.40 ± 0.40^*^	0.012
FE	1.45 ± 0.02	1.42 ± 0.01	1.37 ± 0.01^**^	0.002

*BW* Bod Weight, *ADFI* Average daily feed intake, *ADG* Average daily gain, *FE* Feed efficiency, *CON* Control group (basal diet), *SAN* Sanguinarine group (basal diet containing 0.225 mg/kg sanguinarine), *DHSA* Dihydrosanguinarine group (basal diet containing 0.225 mg/kg dihydrosanguinarine). Values are expressed as mean ± SEM (*n* = 5). ^*^P < 0.05, ^**^P < 0.01 (Compared with the CON group)

### Sanguinarine and dihydrosanguinarine regulated tryptophan metabolism in the intestine

**Fig. 3:**
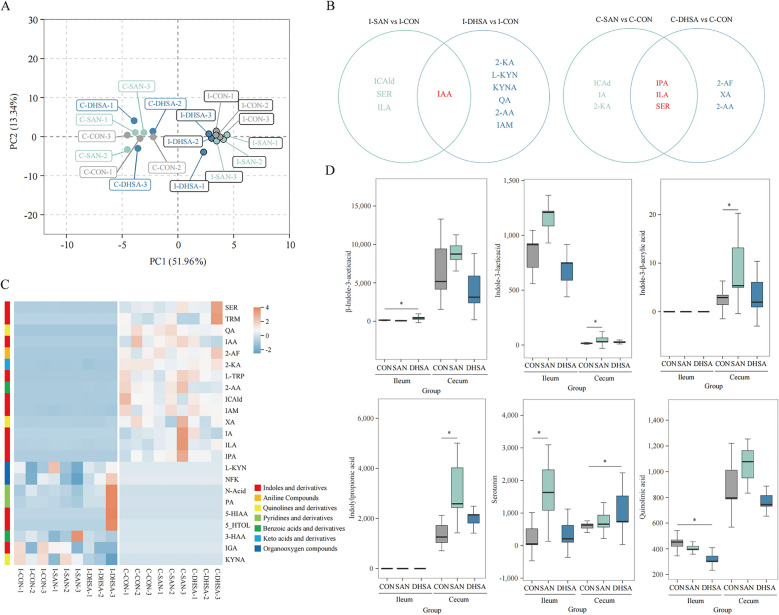
Sanguinarine and dihydrosanguinarine regulated tryptophan metabolism in the intestine. **A** PCA. **B** Venn diagram comparing tryptophan species in the ileum (left) and cecum (right) of broilers between experimental groups and CON group. **C** Heatmap of tryptophan metabolite species. **D** The contents of β-Indole-3-acetic acid, Indole-3-lactic acid, Indole-3-β-acrylic acid, Indolylpropionic acid, serotonin and quinolinic acid in intestinal of broilers. *CON* Control group (basal diet), *SAN* Sanguinarine group (basal diet containing 0.225 mg/kg sanguinarine), *DHSA* Dihydrosanguinarine group (basal diet containing 0.225 mg/kg dihydrosanguinarine). Values are expressed as mean ± SEM (*n* = 3). *P < 0.05; **P < 0.01

The types and contents of tryptophan and its metabolites in the ileum and cecum of broilers were determined using the LC-MS/MS method. As depicted in Fig. 3A, PCA analysis revealed a distinct separation effect between samples from the ileum and cecum of broilers, indicating significant variations in tryptophan composition across different intestinal segments. Furthermore, there was a noticeable segregation in tryptophan compositions between the ileum and cecum of broilers in both the CON group and experimental groups, suggesting that dietary supplementation with SAN and DHSA effectively modulated intestinal tryptophan metabolism in broilers. The Venn diagram reflects the difference metabolites in the intestinal contents of broilers after comparison between the experimental groups and CON group, and the results were shown in Fig. 3B. Compared with the CON group, the ileum of broilers in the SAN group exhibited 4 distinct metabolites, while the DHSA group showed 7. Among these metabolites, β-indole-3-acetic acid was consistently detected across all groups. In terms of cecal composition, both the SAN and DHSA groups displayed 6 different metabolites, with 3 overlapping ones: indole-propionic acid, indole-3-lactic acid, and serotonin. These findings further support that dietary inclusion of SAN and DHSA significantly impacts tryptophan metabolite profiles in the broiler gut. The heat map in Fig. 3C clearly illustrates the predominant tryptophan species present in the gastrointestinal tract of broilers, encompassing indoles and derivatives, aniline compounds, quinolines and derivatives, pyridines and derivatives, benzoic acids and derivatives, keto acids and derivatives, and organooxygen compounds. Tryptophan detected in the gut primarily exists in the form of indole and its derivatives. A total of 12 distinct types were identified, with 10 found in the ileum and 9 in the cecum. In conclusion, SAN and DHSA exert a significant impact on indole tryptophan metabolism upon entering the intestine. Subsequently, differential metabolite analysis was conducted to analyze the indole tryptophan content in the intestine, and the results are presented in Fig. 3D. In comparison to the CON group, both SAN and DHSA exhibited regulatory effects on indole and its derivatives in the intestinal tract. Specifically, SAN significantly increased serotonin levels in the ileum (*P* = 0.035), as well as indole-3-lactic acid, indole-acrylic acid, and indole-propionic acid levels in the cecum (*P* = 0.025, *P* = 0.042 and *P* = 0.039). DHSA supplementation notably elevated indole acetic acid levels in the ileum (*P* = 0.022) and serotonin levels in the cecum (*P* = 0.036). Although DHSA also increased indole-propionic acid levels in the cecum, this difference was not statistically significant (*P* = 0.126). Additionally, we observed that DHSA reduced quinolinic acid levels in the broiler intestines, particularly in the ileum (*P* = 0.032).

### Sanguinarine and dihydrosanguinarine could activate AhR pathway in the intestine

**Fig. 4:**
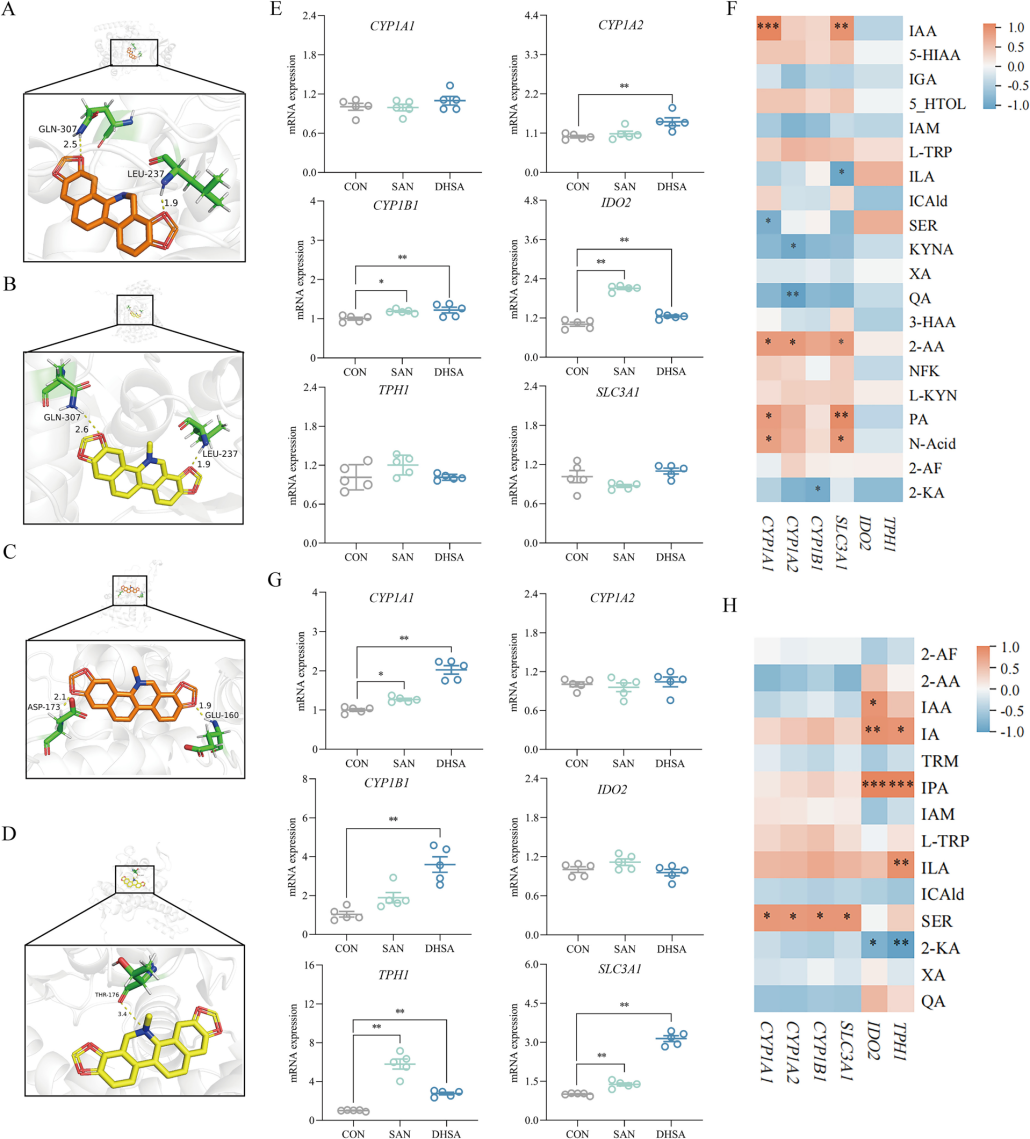
Sanguinarine and dihydrosanguinarine could activate AhR pathway in the intestine. Stereographic and closeup images of **A** sanguinarine and **B** dihydrosanguinarine docked into TPH1 protein and their hydrogen bonding interactions. Stereographic and closeup images of **C** sanguinarine and **D** dihydrosanguinarine docked into CYP1A2 protein and their hydrogen bonding interactions. **E** The relative expression levels of *CYP1A1*, *CYP1A2*, *CYP1B1*, *IDO2*, *TPH1* and *SLC3A1* in the ileum were analyzed by qRT-PCR. **F** To evaluate the correlation between the types and contents of tryptophan and its metabolites and AhR pathway gene expression in ileum of broilers by spearman correlation analysis. **G** The relative expression levels of *CYP1A1*, *CYP1A2*, *CYP1B1*, *IDO2*, *TPH1* and *SLC3A1* in the cecum were analyzed by qRT-PCR. **H** To evaluate the correlation between the types and contents of tryptophan and its metabolites and AhR pathway gene expression in cecum of broilers by spearman correlation analysis. *CON* Control group (basal diet),* SAN* Sanguinarine group (basal diet containing 0.225 mg/kg sanguinarine), *DHSA* Dihydrosanguinarine group (basal diet containing 0.225 mg/kg dihydrosanguinarine). Values are expressed as mean ± SEM (*n* = 5). *P < 0.05; **P < 0.01; ***P < 0.001

To further validate the effects of sanguinarine and dihydrosanguinarine on tryptophan metabolism in broilers are related to AhR pathway, we conducted molecular docking analysis to predict their interaction with specific receptor proteins. As illustrated in Fig. 4A–D and Table 5, sanguinarine and dihydrosanguinarine establish hydrogen bond interactions with specific amino acid residues of TPH1 and CYP1A2 receptor proteins. Notably, the docking binding energies were lower than −5 kcal/mol, indicating strong affinity between these compounds and the receptors. Furthermore, compared to sanguinarine, dihydrosanguinarine exhibited a lower energy requirement for binding to the receptor proteins, suggesting an even stronger binding force. Based on the results of molecular docking, we identified the potential of both sanguinarine and dihydrosanguinarine in regulating the AhR signaling pathway. To further validate their effects on the intestinal AhR pathway in broilers, we selected specific genes within this pathway for qRT-PCR verification, the corresponding results are presented in Fig. 4E and G. Compared to the CON group, the relative mRNA expression levels of *TPH1* (*P* < 0.001 and *P* = 0.002) and *SLC3A1* (*P* = 0.004 and *P* < 0.001) in the cecum of broilers in the SAN group and DHSA group were significantly increased. Dietary sanguinarine significantly elevated the relative mRNA expression levels of *IDO2* (*P* < 0.001) and *CYP1B1* (*P* = 0.020) in the ileum of broilers. Additionally, SAN significantly increased the mRNA expression level of *CYP1A1* (*P* = 0.018) in the cecum. It is noteworthy that dihydrosanguinarine had a more pronounced effect on the AhR pathway in the broiler gut, substantially enhancing the relative mRNA expression levels of *CYP1A2*, *CYP1B1* and *IDO2* in the ileum (*P* = 0.003, *P* = 0.008 and *P* = 0.003). Moreover, dihydrosanguinarine not only significantly increased the mRNA expression level of *CYP1A1* (*P* < 0.001) in the cecum, but also elevated the relative mRNA expression level of *CYP1B1* (*P* < 0.001). To investigate the relationship between tryptophan metabolism and the expression of factors in the AhR pathway in the broiler gut, Spearman correlation analysis was employed to assess the association between tryptophan and its metabolites in both the ileum and cecum of broilers and gene expression in the AhR pathway. The corresponding results are presented in Fig. 4F and H. In the ileum, we observed a positive correlation between the expression levels of *CYP1A1* and *SLC3A1* and the concentrations of β-indole-3-acetic acid, 2-aminobenzoic acid, picolinic acid and nicotinic acid. Additionally, the expression levels of *CYP1A2* were positively associated with 2-aminobenzoic acid, but negatively correlated with quinolinic acid and kynurenic acid. In the cecum, the expression levels of *IDO2* and *TPH1* exhibited a positive correlation with the contents of indole-3-β-acrylic acid and indolylpropionic acid, while displaying a negative correlation with the contents of 2-ketoadipic acid. Furthermore, the expression levels of *TPH1* were positively associated with indole-3-lactic acid, *IDO2* expression showed a positive relationship with β-indole-3-acetic acid, and the expression levels of *CYP1A1*, *CYP1A2*, *CYP1B1* and *SLC3A1* were positively associated with serotonin. In summary, sanguinarine and dihydrosanguinarine can activate the AhR pathway and regulate the expression of related genes in this pathway by modulating tryptophan metabolism in the broiler gut.

**Table 5:** Molecular docking parameters

Item	Binding energy, kcal/mol	Number of hydrogen bond
SAN-TPH1	−6.28	2
DHSA-TPH1	−6.67	2
SAN-CYP1A2	−5.57	2
DHSA-CYP1A2	−5.79	1

*SAN* Sanguinarine, *DHSA* Dihydrosanguinarine, *TPH1* Tryptophan hydroxylase 1, *CYP1A2* Cytochrome P450, family 1, subfamily A, polypeptide 2

### Sanguinarine and dihydrosanguinarine regulated the intestinal flora structure and abundance

**Fig. 5:**
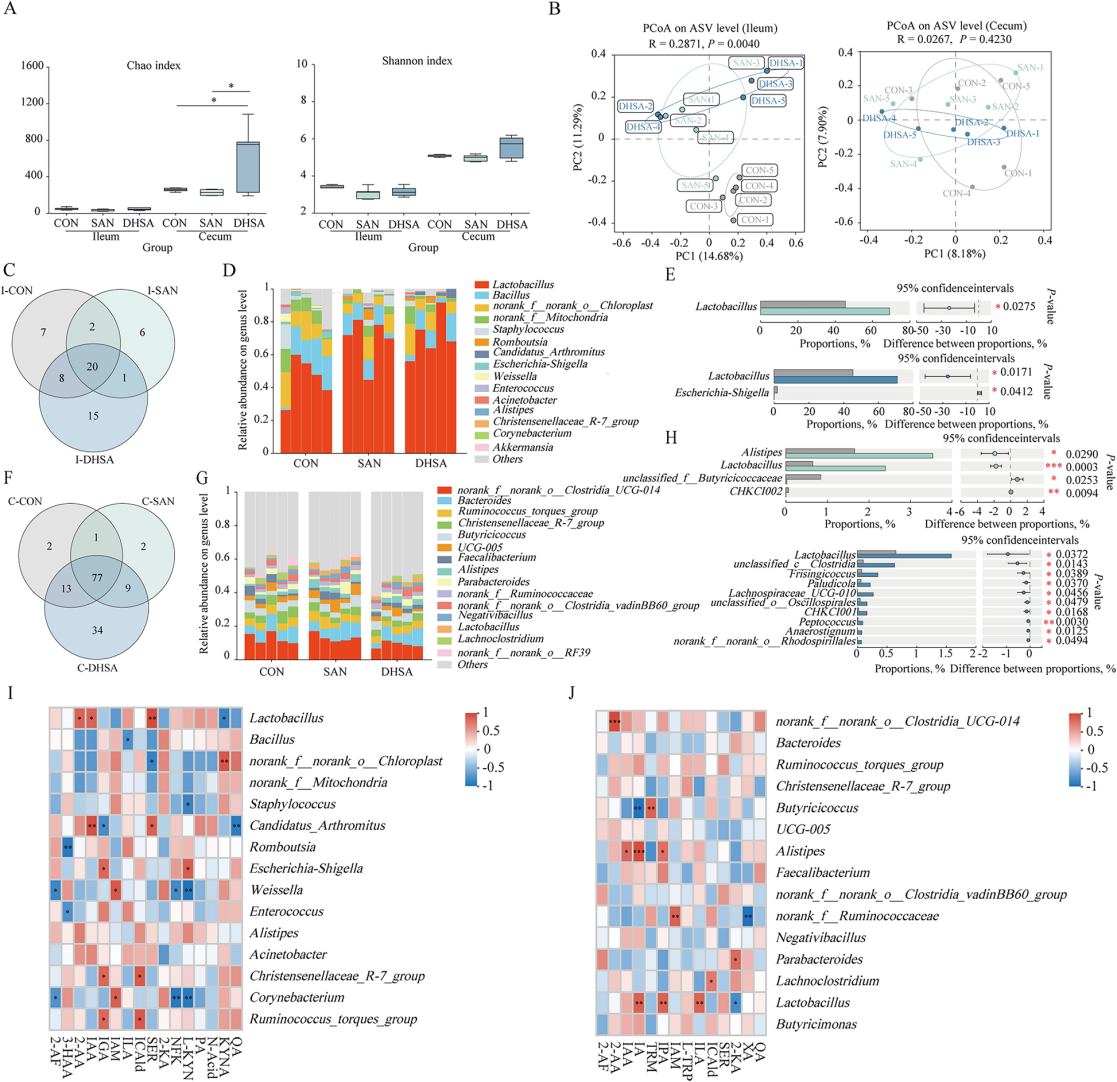
Sanguinarine and dihydrosanguinarine regulated the intestinal flora structure and abundance. **A** Evaluation of α diversity of the intestinal microbiota in broilers. **B** PCoA plot of the ileum (left) and cecum (right) microbiota of broilers. **C** Venn diagram analysis of ileum flora in broilers. **D** Composition of the top 15 abundant microorganisms in ileal of broilers based on genus level. **E** The difference of microflora abundance in ileum content samples between experimental groups and CON group. **F** Venn diagram analysis of cecum flora in broilers. **G** Composition of the top 15 abundant microorganisms in cecal of broilers based on genus level. **H** The difference of microflora abundance in cecum content samples between experimental groups and CON group. **I** Correlation analysis between the top 15 abundant microorganisms in the ileum of broilers and the contents of tryptophan and its metabolites based on genus level. **J** Correlation analysis between the top 15 abundant microorganisms in the cecum of broilers and the contents of tryptophan and its metabolites based on genus level. CON Control group (basal diet), SAN Sanguinarine group (basal diet containing 0.225 mg/kg sanguinarine), DHSA Dihydrosanguinarine group (basal diet containing 0.225 mg/kg dihydrosanguinarine). Values are expressed as mean ± SEM (*n* = 5). *P < 0.05; **P < 0.01; ***P < 0.001

To further investigate the potential mechanism by which sanguinarine and dihydrosanguinarine regulate tryptophan metabolism in broilers, we performed 16S rRNA microbiome sequencing on the intestinal contents of broilers from each experimental group. As depicted in Fig. 5A, compared to the CON group, the Chao index (*P* = 0.126 and *P* = 0.132) and Shannon index (*P* = 0.146 and *P* = 0.135) of ileal and cecal microbiota in broilers from the SAN group were decreased. However, dietary supplementation with DHSA significantly increased both the Chao index (*P* = 0.038) and Shannon index of cecal microbiota, indicating its potential to modulate the intestinal flora structure of broilers. The PCoA analysis further revealed the variations in intestinal microbial communities among different groups, as illustrated in Fig. 5B. Both SAN and DHSA significantly influenced the composition of ileal microbiota compared to the CON group (*P* = 0.004). Although these two additives affected the structure and abundance of cecal microbiota, the differences were not statistically significant (*P* = 0.423). The intestinal flora composition of broilers in each group was analyzed using a Venn diagram, and the results were presented in Fig. 5C and F. At the genus level analysis, there were 20 and 77 shared OTUs in the ileum and cecum microbiota, respectively. Compared to the CON group, the SAN group had 7 unique OTUs in the ileum, while the DHSA group had 16. In terms of cecal flora, the SAN group had 11 unique OTUs, and the DHSA group had 43. These findings suggest that DHSA exerts a more pronounced impact on the structure and abundance of intestinal flora in broilers. Through community histogram analysis, we directly observed changes in the microbial composition of each experimental group compared to the CON group. As shown in Fig. 5D and G, at the genus level, both SAN and DHSA increased the relative abundance of *Lactobacillus* in the ileum and cecum of broilers. Additionally, SAN and DHSA increased the relative abundance of *Candidatus_Arthromitus* and *Romboutsia* specifically in the ileum. In the cecum, the relative abundance of *Alistipes* increased, while that of *Escherichia-Shigella* decreased in the ileum. Furthermore, *Christensenellaceae_R-7_group* and *Butyricicoccus* showed reduced abundance in the cecum due to these additives. Based on the genus level analysis, the significant differences in dominant bacterial genera between ileal and cecal microbiota of broilers in each group were assessed, and the results are presented in Fig. 5E and H. Compared to the CON group, the SAN group exhibited a significant increase in *Lactobacillus* abundance in the ileum (*P* = 0.027). Furthermore, DHSA supplementation not only significantly increased *Lactobacillus* abundance in the ileum (*P* = 0.017), but also significantly reduced the relative abundance of *Escherichia-Shigella* (*P* = 0.041). In the cecum, both SAN (*P* < 0.001) and DHSA (*P* = 0.037) significantly increased the relative abundance of *Lactobacillus*. Given the significant influence of SAN and DHSA on *Lactobacillus* abundance in the gut, we conducted in vitro experiments to examine their effects on *Lactobacillus* growth. As shown in Fig. S2, varying doses of SAN and DHSA effectively lowered the pH of the bacterial solution by increasing the concentration of short-chain fatty acids, thereby creating a more favorable acidic environment for the proliferation of *Lactobacillus reuteri*. The Spearman correlation analysis was employed to investigate the association between the content of tryptophan and its metabolites in the ileum and cecum, as well as the abundance of the microbiota. The results are presented in Fig. 5I and J. In the ileum, the relative abundance of *Lactobacillus* positively correlated with serotonin, 2-aminobenzoic acid and β-indole-3-acetic acid, but negatively correlated with kynurenic acid. The relative abundance of *Escherichia-Shigella* positively correlated with 3-indoleglyoxylic acid and L-kynurenine. In the cecum, the relative abundance of *Lactobacillus* positively correlated with indole-3-lactic acid, indolylpropionic acid and indole-3-β-acrylic acid, but negatively correlated with 2-ketoadipic acid. Additionally, the abundance of *Alistipes* positively correlated with β-indole-3-acetic acid, indolylpropionic acid and indole-3-β-acrylic acid.

### Sanguinarine and dihydrosanguinarine could enhance the immune function of cecum

**Fig. 6:**
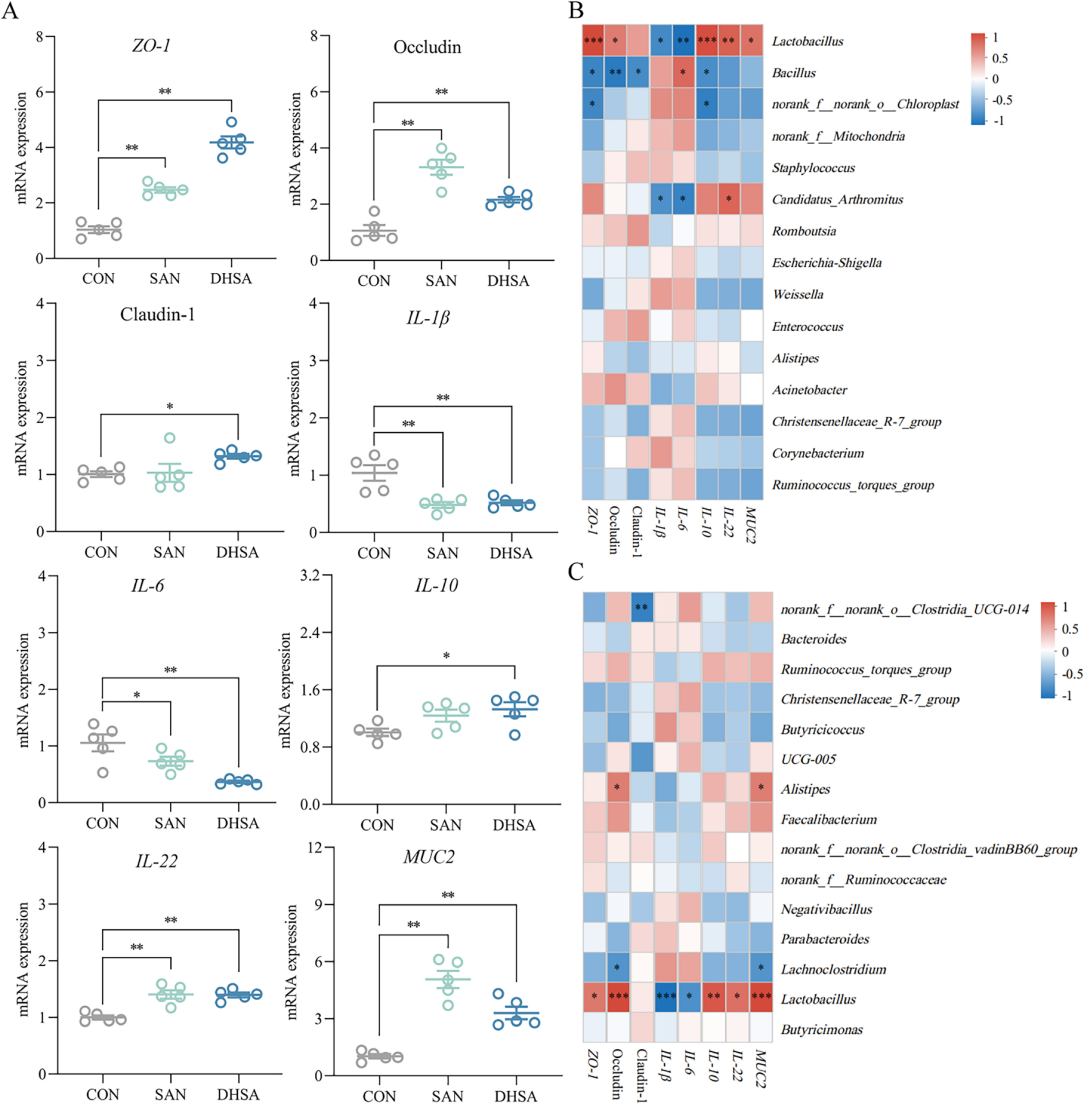
Sanguinarine and dihydrosanguinarine could enhance the immune function of cecum. **A** The relative expression levels of *ZO-1*, occludin, claudin-1, *MUC2*, *IL-1β*, *IL-6,* *IL-10 *and* IL-22* in the cecum were analyzed by qRT-PCR. **B** Correlation analysis between the top 15 abundant microorganisms in the ileum of broilers and the contents of ileum immune factor based on genus level. **C** Correlation analysis between the top 15 abundant microorganisms in the cecum of broilers and the contents of cecum immune factor based on genus level. *CON* Control group (basal diet), *SAN* Sanguinarine group (basal diet containing 0.225 mg/kg sanguinarine), *DHSA* Dihydrosanguinarine group (basal diet containing 0.225 mg/kg dihydrosanguinarine). Values are expressed as mean ± SEM (*n* = 5). *P < 0.05; **P < 0.01; ***P < 0.001

As shown in Fig. 6A, compared to the CON group, the experimental groups exhibited significantly increased mRNA expression levels of *ZO-1* (*P* < 0.001 and *P* < 0.001), occludin (*P* < 0.001 and *P* = 0.002), *MUC2* (*P* < 0.001 and *P* < 0.001) and *IL-22* (*P* < 0.001 and *P* < 0.001) in the cecal tissue of broilers. In contrast, the expression of *IL-1β* (*P* < 0.001 and *P* = 0.001) showed a significant decrease. Furthermore, the SAN group exhibited a notable reduction in *IL-6* content in the cecum of broilers (*P* = 0.038). Notably, dietary supplementation with DHSA had more pronounced effects on immune-related factors in the cecum, significantly increasing the contents of claudin-1 (*P* = 0.044) and *IL-10* (*P* = 0.015) while significantly decreasing *IL-6* content (*P* < 0.001). Spearman correlation analysis was performed to explore the association between intestinal microbiota and mRNA expression levels of immune-related factors in broilers, and the findings were presented in Fig. 6B and C. The relative abundance of *Lactobacillus* exhibited a positive correlation with the expression of *ZO-1*, occludin, *IL-10*, *IL-22* and *MUC2*. Conversely, it displayed a negative correlation with the levels of *IL-1β* and *IL-6* in intestinal tissue. Furthermore, the relative abundance of *Bacillus* in the ileum demonstrated a positive correlation with *IL-6* expression but a negative correlation with the expression of *ZO-1*, occludin, claudin-1, and *IL-10*. In the cecum, the abundance of *Alistipes* exhibited a positive correlation with the concentrations of occludin and *MUC2*.

## Discussion

Research has shown that dietary inclusion of berberine (600 mg/kg) significantly enhances hepatic function, decreases feed conversion ratio (FCR), and improves growth performance in broilers [19]. Notably, dihydroberberine, a metabolite of berberine, exhibits a physiological structure and pharmacological effects that closely resemble those of berberine. However, dihydroberberine demonstrates higher bioavailability than berberine, leading to more significant therapeutic outcomes [20]. Sanguinarine and the berberine are two structurally similar plant alkaloids [21]. Inspired by these findings, we hypothesized that dihydrosanguinarine, as a metabolite of sanguinarine, might also enhance broiler growth performance when included in feed, potentially even more effectively than sanguinarine itself. Our study confirmed this hypothesis, showing that dietary supplementation with either sanguinarine or dihydrosanguinarine increased average daily gain and reduced feed efficiency values in broilers. Notably, dihydrosanguinarine exerted a more pronounced growth-promoting effect compared to sanguinarine. Previous research has demonstrated that improved growth performance is often associated with enhanced immune function [22]. Based on this, we propose the hypothesis that the metabolic conversion of sanguinarine to dihydrosanguinarine may augment intestinal immune function, thereby ultimately improving growth performance in broilers. Subsequent studies will further investigate the specific effects of dihydrosanguinarine on intestinal immune function in broilers.

The integrity of the intestinal barrier function is essential for the normal physiological function of the gut. It has been reported that dietary supplementation with sanguinarine can ameliorate high-fat diet induced dysfunction in the physical barrier of the intestine in grass carp by upregulating the expression levels of tight junction protein-related genes, increasing intestinal villus height and enhancing goblet cell count [23]. Similarly, dietary sanguinarine significantly reduces ileal crypt depth, increases the villus height-to-crypt depth ratio, improves intestinal morphological structure, and consequently enhances broiler growth performance [24, 25]. As a metabolic derivative of berberine, dihydroberberine has been shown to enhance the intestinal barrier function by blocking the TLR4/MyD88/NF-κB signaling pathway, reducing the levels of pro-inflammatory cytokines in the colon, and upregulating the expression levels of intestinal tight junction proteins and mucins, thereby effectively alleviating dextran sulfate sodium-induced ulcerative colitis. Notably, dihydroberberine exhibits a more pronounced effect compared to berberine [7]. In this study, dietary supplementation with sanguinarine and dihydrosanguinarine were found to increase villus height and the villus height-to-crypt depth ratio in the ileum of broilers, while reducing the crypt depth, thus helping to preserve intestinal morphology. Additionally, both compounds increased the relative mRNA expression levels of tight junction proteins in the ileum and cecum of broilers. To summarize, as a major metabolite of sanguinarine, dihydrosanguinarine effectively enhances the intestinal physical barrier function, preserves normal intestinal tissue structure, and improves the intestinal immune function in broilers.

The synthesis and secretion of immune-related factors are essential for maintaining intestinal immune function [26]. Previous studies have demonstrated that heat stress induces the upregulation of inflammatory factors in intestinal tissue, such as IL-1β and IL-6, while simultaneously reducing the expression of the anti-inflammatory factor IL-10. This dysregulation exacerbates intestinal inflammation, impairs mucosal barrier function, and ultimately compromises intestinal health [27]. The reported findings indicate that sanguinarine can attenuate the expression of inflammatory factors in the small intestine of rats, including IL-1β, IL-6 and TNF-α. This inhibition effectively suppresses the inflammatory response and mitigates tissue damage [28, 29]. Dihydrosanguinarine, a metabolite of sanguinarine, exhibits comparable effects by inhibiting the inflammatory response [30]. Anti-inflammatory factors such as IL-10 and IL-22 play a crucial role in maintaining the homeostasis of the intestinal mucosa [31]. Secretory immunoglobulin A (SIgA), a prominent marker of mucosal immune status, is essential for protecting the gut from antigen invasion and promoting intestinal health [32, 33]. Studies have shown that the *Macleaya cordata* extract enhances the levels of IL-10, TGF-β, IgG and SIgA in neonatal piglets. It also increases the relative mRNA expression levels of *TGF-β* and *IL-10* in ileal mucosa, thereby alleviating inflammation, improving immune function, decreasing diarrhea incidence, and ultimately enhancing growth performance [34]. In this experiment, dietary supplementation with sanguinarine and dihydrosanguinarine significantly reduced IL-1β content and increased the concentrations of IL-22 and SIgA in ileal tissue. However, compared to the sanguinarine group, dietary supplementation with dihydrosanguinarine further decreased IL-6 concentration and increased IL-10 levels in the ileum of broilers. Additionally, both additives increased the relative mRNA expression levels of *IL-10* and *MUC2* in ileal tissue, with dihydrosanguinarine showing more pronounced effects. Furthermore, these additives increased the relative mRNA expression levels of *MUC2* and *IL-22* in cecum while significantly decreasing the mRNA expression levels of *IL-1β* and *IL-6* in tissues. Notably, dihydrosanguinarine had a more significant effect on regulating intestinal immune function than sanguinarine based on our comparison results between the two groups. These findings further support our previous hypothesis that dihydrosanguinarine is the critical metabolite for regulating intestinal immunity.

Growing evidence indicates that the gut microbiota synthesizes tryptophanase through metabolic processes, facilitating the conversion of 4%-6% of tryptophan into indole and its derivatives. These compounds play a key role in enhancing intestinal immune function, thereby contributing to the maintenance of optimal gut health [35–38]. As a natural immune modulator, indole increases the mRNA expression levels of tight junction protein, such as *ZO-1* and claudins, in intestinal epithelial tissue. This mechanism helps to maintain the integrity of the intestinal barrier and enhances intestinal immune function [39]. In this study, dietary supplementation with sanguinarine and dihydrosanguinarine effectively modulated tryptophan metabolism in the intestinal tract of broilers, with a particularly pronounced effect on indole and its derivatives compared to other tryptophan metabolites. For instance, indolylpropionic acid can attenuate the expression of lipopolysaccharide-induced inflammatory factors by enhancing trans-epithelial resistance in intestinal epithelial cells and reducing paracellular permeability in the colon, thereby improving intestinal barrier function [40]. β-Indole-3-acetic acid enhances intestinal immune function by inhibiting the production of pro-inflammatory cytokines TNF-α and IL-6, while increasing the concentration of anti-inflammatory factor IL-10 [41]. Serotonin, by binding to its specific receptors, activates immune cells such as T cells, dendritic cells, and macrophages, inducing them to secrete downstream cytokines and thereby enhancing the immune response [42]. Indole-3-β-acrylic acid has been shown to decrease intestinal permeability, subsequently reducing interleukin secretion in colonic innate lymphoid cells and aiding in mitigating infection-induced intestinal damage [43]. Additionally, indole-3-lactic acid has been shown to inhibit colorectal cancer progression by suppressing RORγt transcriptional activity, leading to a reduction in the helper T cell population [44]. In our study, dietary supplementation with sanguinarine significantly increased serotonin levels in the ileum of broilers and elevated the levels of indole-3-lactic acid, indole-3-β-acrylic acid and indolylpropionic acid in the cecum. In contrast, dihydrosanguinarine significantly increased the contents of β-indole-3-acetic acid in the ileum and serotonin in the cecum of broilers. It is noteworthy that apart from modulating indoles and their derivatives, we also observed that dihydrosanguinarine effectively reduced quinolinic acid levels in the gut of broilers, particularly in the ileum. Quinolinic acid, known for its neurotoxic effects, is associated with neuroinflammation and excitatory toxicity [45]. In summary, the upregulation of indole and its derivatives in the intestinal tract by sanguinarine and dihydrosanguinarine effectively modulates the expression of inflammatory factors, thereby enhancing intestinal immune function.

Previous studies have demonstrated that indole and its derivatives can activate the AhR and form heterodimers with its nuclear transcription factor (ARNT) [46]. Subsequently, this dimer complex translocates to the promoter region of AhR target genes, thereby inducing the expression of immunomodulatory genes and regulating intestinal immune function [47–49]. The activation of AhR is closely associated with the expression of inflammatory cytokines and the production of cytochrome P450 genes, which play a crucial role in maintaining host homeostasis [50]. Reports indicate that the expression of the indoleamine 2,3-dioxygenase (IDO) gene facilitates intestinal epithelial cell differentiation and mucus secretion, contributing to the maintenance of intestinal homeostasis [51]. Additionally, the enzyme tryptophan hydroxylase (TPH) catalyzes the enzymatic conversion of tryptophan into 5-hydroxytryptophan, thereby promoting intestinal growth and maintaining the integrity of the intestinal mucosal barrier [52]. Based on previous transcriptomic data, we identified that dietary sanguinarine modulates the expression of several genes associated with the intestinal AhR pathway. qRT-PCR validation further confirmed that dietary supplementation with sanguinarine and dihydrosanguinarine significantly increase the relative mRNA expression levels of *CYP1B1* and *IDO2* in the ileum of broilers. Additionally, the relative mRNA expression levels of *CYP1A1*, *SLC3A1* and *TPH1* were increased in the cecum of broilers. Notably, compared to the sanguinarine group, dihydrosanguinarine further enhanced the mRNA expression levels of *CYP1A2* in the ileum and *CYP1B1* in the cecum. In summary, these findings suggest that sanguinarine and dihydrosanguinarine enhance intestinal immune function by regulating indole tryptophan metabolism and modulating gene expression within the AhR pathway. However, it should be emphasized that dihydrosanguinarine exhibits a more pronounced effect on regulating AhR pathway-related gene expression than sanguinarine. Previous studies have indicated that gut microbiota metabolize tryptophan into indole derivatives, thereby strengthening host immunity [53]. Therefore, to further investigate the microecological regulatory mechanism of dihydrosanguinarine in tryptophan metabolism, we examined its impact on the structure and abundance of intestinal microbiota in broilers.

The intestinal microbiota has been reported to modulate host intestinal morphology and physiological function, thereby augmenting the intestinal immune response and promoting overall host health [54]. Extracts of *Macleaya cordata* have demonstrated the ability to enhance the abundance of *Lactobacillus*, reduce levels of *Escherichia-Shigella*, and stimulate the production of SCFAs such as acetate, propionate and butyrate. These SCFAs contribute to maintaining normal intestinal morphology and enhancing immune function [55, 56]. Previous research has shown that dietary supplementation with 50 mg/kg sanguinarine modulates the structure of the gut microbiota in grass carp, offering anti-inflammatory and antioxidant effects while reducing damage to the intestinal barrier induced by a high fat diet [57]. The present study demonstrates that dietary supplementation with sanguinarine and dihydrosanguinarine regulates broiler gut microbiota structure and abundance, significantly increasing the relative abundance of *Lactobacillus*. Furthermore, dihydrosanguinarine notably reduced *Escherichia-Shigella* colonization in the ileum of broilers. Based on these findings, we hypothesize that sanguinarine and dihydrosanguinarine exhibit similarities in modulating tryptophan metabolism within the broiler intestine, potentially attributed to their shared influence on promoting *Lactobacillus* proliferation. Subsequently, we further demonstrated through in vitro experiments that *Lactobacillus* serves as the target bacterial genus for sanguinarine and dihydrosanguinarine. The findings revealed that these compounds effectively stimulate the production of short-chain fatty acids in bacterial solution, leading to a reduction in pH levels and creating an acidic environment conducive to the proliferation of *Lactobacillus reuteri*. Increasing evidence suggests that *Lactobacillus reuteri* can modulate tryptophan metabolism, enhance indole and derivatives, activate the AhR pathway, and subsequently regulate inflammatory factors, tight junction proteins, and intestinal barrier integrity, thereby providing anti-inflammatory benefits [58, 59]. In this study, we investigated the correlation between intestinal microbiota composition, tryptophan metabolism and intestinal immune factors. The findings revealed that increasing the relative abundance of *Lactobacillus* in the intestinal tract promotes tryptophan metabolism and enhances the contents of indole and its derivatives. This subsequently activates the AhR pathway, regulates downstream factor expression, inhibits inflammatory factor expression, and elevates anti-inflammatory factor levels. Additionally, these changes facilitate the mRNA expression of intestinal tight junction proteins, ultimately leading to enhanced intestinal immune function.

## Conclusions

In summary, upon entering the body, sanguinarine undergoes metabolism and is converted into dihydrosanguinarine, which exhibits enhanced bioavailability. This metabolic transformation facilitates the proliferation of *Lactobacillus*. Under the positive influence of *Lactobacillus*, tryptophan metabolism is redirected toward the production of indoles and their derivatives, which further activate the AhR signaling pathway in the intestine. By regulating the expression of key factors in this pathway, there is a significant inhibition of the inflammatory factors *IL-1β* and *IL-6* in intestinal tissue, while promoting the anti-inflammatory factors *IL-10* and *IL-22*. Additionally, this process upregulates the expression of tight junction proteins, thereby enhancing intestinal barrier function and elevating the expression levels of immune-related factors. These effects collectively boost intestinal immune function and improve broiler growth performance.

## Data Availability

The ileum transcriptome sequencing data that were generated and analyzed during the current study are available in the NCBI primary data archive (PDA) with accession number PRJNA1043866. These data can be found here: http://www.ncbi.nlm.nih.gov/bioproject/1043866. The 16S rRNA gene sequencing data that were generated and analyzed during the current study are available in the NCBI primary data archive (PDA) with accession number PRJNA1181637. These data can be found here: http://www.ncbi.nlm.nih.gov/bioproject/1181637.

## Abbreviations

ADFI: Average daily feed intake
ADG: Average daily gain
AhR: Aryl hydrocarbon receptor
AhRR: Aryl-hydrocarbon receptor repressor
ARNT: Aryl hydrocarbon receptor nuclear translocator
CYP1A1: Cytochrome P450, family 1, subfamily A, polypeptide 1
CYP1A2: Cytochrome P450, family 1, subfamily A, polypeptide 2
CYP1B1: Cytochrome P450, family 1, subfamily B, polypeptide 1
DHSA: Dihydrosanguinarine
ELISA: Enzyme-linked immunosorbent assay
FC: Fold Change
FE: Feed efficiency
H&E: Hematoxylin and eosin
HSP90B1: Heat shock protein 90 beta family member 1
IDO2: Indoleamine 2,3-dioxygenase 2
IL-1β: Interleukin 1 beta
IL-6: Interleukin 6
IL-10: Interleukin 10
IL-22: Interleukin 22
MUC2: Mucin 2
NQO1: NAD(P)H quinone dehydrogenase 1
qRT-PCR: Quantitative real-time PCR
SAN: Sanguinarine
SCFA: Short-chain fatty acid
SIgA: Secreted immunoglobulin A
SLC3A1: Solute carrier family 3 member 1
TPH1: Tryptophan hydroxylase 1
TPH2: Tryptophan hydroxylase 2
UGT1A1: UDP Glucuronosyltransferase family 1 member A1
ZO-1: Zonula occludens-1
